# *NGFR* Gene and Single Nucleotide Polymorphisms, rs2072446 and rs11466162, Playing Roles in Psychiatric Disorders

**DOI:** 10.3390/brainsci12101372

**Published:** 2022-10-09

**Authors:** Longyou Zhao, Binyin Hou, Lei Ji, Decheng Ren, Fan Yuan, Liangjie Liu, Yan Bi, Fengping Yang, Shunying Yu, Zhenghui Yi, Chuanxin Liu, Bo Bai, Tao Yu, Changqun Cai, Lin He, Guang He, Yi Shi, Xingwang Li, Shaochang Wu

**Affiliations:** 1 Lishui No.2 People’s Hospital, 69 Beihuan Road, Liandu District, Lishui 323000, China; 2 Bio-X Institutes, Key Laboratory for the Genetics of Developmental and Neuropsychiatric Disorders, Shanghai Jiao Tong University, 1954 Huashan Road, Shanghai 200030, China; 3 Shanghai Key Laboratory of Psychotic Disorders, and Brain Science and Technology Research Center, Shanghai Jiao Tong University, 1954 Huashan Road, Shanghai 200030, China; 4 School of Mental Health, Jining Medical University, 16 Hehua Road, Taibaihu New District, Jining 272067, China; 5 Wuhu Fourth People’s Hospital, 1 Xuxiashan Road, Wuhu 241002, China

**Keywords:** psychiatric disorders, schizophrenia, major depressive disorders (MDD), association analyses, *NGFR* gene, p75 neurotrophin receptor, shRNA lentivirus, knock down, social behavior, functional study

## Abstract

Psychiatric disorders are a class of complex disorders characterized by brain dysfunction with varying degrees of impairment in cognition, emotion, consciousness and behavior, which has become a serious public health issue. The *NGFR* gene encodes the p75 neurotrophin receptor, which regulates neuronal growth, survival and plasticity, and was reported to be associated with depression, schizophrenia and antidepressant efficacy in human patient and animal studies. In this study, we investigated its association with schizophrenia and major depression and its role in the behavioral phenotype of adult mice. Four *NGFR* SNPs were detected based on a study among 1010 schizophrenia patients, 610 patients with major depressive disorders (MDD) and 1034 normal controls, respectively. We then knocked down the expression of *NGFR* protein in the hippocampal dentate gyrus of the mouse brain by injection of shRNA lentivirus to further investigate its behavioral effect in mice. We found significant associations of s2072446 and rs11466162 for schizophrenia. *Ngfr* knockdown mice showed social and behavioral abnormalities, suggesting that it is linked to the etiology of neuropsychiatric disorders. We found significant associations between *NGFR* and schizophrenia and that *Ngfr* may contribute to the social behavior of adult mice in the functional study, which provided meaningful clues to the pathogenesis of psychiatric disorders.

## 1. Introduction

Psychiatric disorders are a class of complex disorders characterized by brain dysfunction with varying degrees of impairment in cognition, emotion, consciousness and behavior, including schizophrenia and major depressive disorders (MDD). Psychiatric disorders are becoming a serious public health issue due to their first rank in disease burden among non-fatal diseases worldwide. Previous genetic studies, including twin, adoption and family studies [1,2,3], showed that the heritability of psychiatric disorders was around 80%, and the gene-environment interactions played an important role in their pathogenesis.

Evidence from previous research implicated that Corpus Callosum (CC) white matter tracts deficits, dissociative symptoms and abnormal fatty acid (FAs) metabolism relates to schizophrenia [4,5,6]. Recent studies found that mitochondrial functions compromise influenced by the loss of mitochondrial stress resilience, activation of the tryptophan (Trp)–kynurenine (KYN) metabolic system and vitamin D deficiency may contribute to the development of psychiatric disorders [7,8]. Some other researchers found that lipophilic statins (including simvastatin), bioactive kynurenines and their analogs can be neuroprotective agents [9,10]. It was reported that synaptic plasticity defects were possibly due to neurodevelopmental and neurodegenerative abnormalities and could contribute to cognitive impairment underlying schizophrenia and some other psychiatric disorders [11]. Synaptic plasticity regulation genes, especially the ones that encode neurotrophic factor and their receptors, are thus believed to be involved in neuronal development, synapse generation, and response to stress/anxiety stimuli [12,13] and may have important roles in the molecular mechanism of cognition impairment in schizophrenia, MDD and some other psychiatric disorders, including nerve growth factor receptor (*NGFR*).

The *NGFR* gene encodes the p75 neurotrophin receptor (p75NTR), which participates in the controlling of many signaling pathways by interacting with all kinds of TRK receptors, sorting proteins and NOGO receptors. Previous research showed that *NGFR* is involved in neurogenesis, regulation of sprouting, synaptogenesis and pruning, which contributes to altered neural functions, and is thought to be the basis of psychiatric disorders [14,15,16]. Studies showed that the serum *NGFR* levels in patients with depression [17,18], schizophrenia [19] and bipolar disorder [20] were significantly different from those in healthy controls. *NGFR* gene polymorphisms were reported to be associated with depression, schizophrenia and antidepressant efficacy [21,22]. Increasing evidence based on animal studies found that genetic variants in *NGFR* could alter the brain’s susceptibility to psychiatric disorders. For instance, a study showed that in the brain of rats with unpredictable chronic mild stress (UCMS), the apoptosis signal of proBDNF/*NGFR*/sorting protein was activated, and the mRNA and protein expression levels of *NGFR* were increased [23]). *Ngfr*-knockout mice exhibited or alleviated behavioral deficits such as anxiety, spatial memory impairment and depression [24,25,26]. It was reported that the *NGFR* SNPs rs11466155 and rs734194 were strongly associated with schizophrenia based on the Armenia Caucasus populations [19].

In view of a series of research above indicating that the *NGFR* gene may be closely related to the pathogenesis of psychiatric disorders such as depression and schizophrenia, we performed an association study of the Chinese Han population to investigate the role of the four *NGFR* SNPs (rs575791, rs1035050, rs2072446, rs11466162, Table 1) in schizophrenia and MDD. Furthermore, we knocked down the expression of *Ngfr* in the hippocampal dentate gyrus of adult mice brain by injection of shRNA lentivirus to examine the behavioral alterations it may cause in mice.

## 2. Materials and Methods

### 2.1. Participants and Animals

In this research, 1010 schizophrenic patients (396 females and 614 males, age: 43.04 ± 12.91, onset age: 25.93 ± 9.62) and 1034 healthy controls (446 females and 588 males, age: 34.06 ± 10.00) were recruited from Chinese Han population. All patients were diagnosed on the basis of DSM-IV criteria by two independent, experienced psychiatrists in two separate clinical interviews. All controls were in good health, and none of them showed symptoms of any psychiatric disorders.

In order to analyze the candidate site of major depression, we recruited 610 unrelated major depressive disorder patients (324 females and 286 males, age: 36.31 ± 12.10) and used the same 1034 individuals as healthy controls. Each of the patients was strictly diagnosed by two experienced psychiatrists adopting DSM-IV criteria independently. The degree of depression was assessed by a 17-item Hamilton Depression Rating Scale (HAMD). Patients pregnant with other psychiatric disorders and substance abuse were excluded.

All the participants were of unrelated Chinese Han origin. All of them signed the informed consent, and the study was appraised and confirmed by the Ethics Committee of the Bio-X center, Shanghai Jiao Tong University (M16033, 2016). Six- to eight-week-old male mice (C57BL/6) were obtained from Shanghai Lingchang Biotech Co., Ltd. All experiments were performed using standard protocols and approved by the Animal Care and Ethics Committee of Shanghai Jiao Tong University, China (202006003, 2020).

### 2.2. DNA Extraction and Genotyping 

In the present study, we collected peripheral venous blood from each participant and applied AxyPrep Blood Genomic DNA Miniprep Kit to DNA extractions. *NGFR* SNPs were selected from the HapMap database (release #24) of the Chinese Han population (minor allele frequency, MAF ≥ 0.05). The detailed information on SNPs is summarized in Table 1. Four SNPs (rs575791, rs1035050, rs2072446, rs11466162) were genotyped by MassARRAY^®^ Analyzer platform (Agena, San Diego, CA, USA). All the probes and primers were designed by My-sequenom online software Assay Design Suite v2.0 (Agena, San Diego, CA, USA). Each tube in the polymerase chain reaction contained 10 ng genomic DNA dissolved in 5 μL buffer.

### 2.3. Statistical Analysis 

For all analyses, we set statistical significance at a *p*-value < 0.05. In this study, we analyzed the allelic and genotypic distributions, Hardy–Weinberg equilibrium and pairwise linkage disequilibrium (LD) by SHEsis (http://analysis.bio-x.cn/myAnalysis.php) (accessed on 24 February 2022). Comparisons of allele and genotype frequencies between cases and controls were performed through the Chi-square test. Linkage disequilibrium of the four pairs of SNPs within *NGFR* was measured by standardized D’. The association between the candidate SNPs with the MDD/schizophrenia risk in five genetic models (codominant, dominant, recessive, over-dominant and log-additive models, respectively) was assessed by “genetic” packages in R software (version 4.1.3., accessed on 10 March 2022, https://www.r-project.org/) with the odds ratios (ORs) and their 95% confidence intervals (CIs). Pairwise linkage disequilibrium (LD) and haplotype constitution were tested using Haploview 4.2 (accessed on 15 March 2022).

### 2.4. Behavioral Tests on Ngfr-Knock-Down Mice

#### 2.4.1. Construction of *Ngfr*-Interference Plasmid

We designed and selected three shRNA with the highest scores on the Invitrogen website (https://rnaidesigner.thermofisher.com/) (accessed on 25 June 2022) for mice *Ngfr*-interference plasmid construction and subsequent interference efficiency verification. The plasmid skeleton was obtained from Li Weidong’s research group at Shanghai Jiao Tong University, and the restriction enzyme cutting sites used were BamHI and XbaI. The control plasmid and the inserted control sequence were proved not to interfere with any known genes after whole-genome alignment in mice. The DNA sequence was synthesized by Shanghai Jieli Bio-Technology Co., LTD. ShRNA and control sequences are shown in Table 2 (5′-3′ direction).

#### 2.4.2. Construction of *Ngfr*-Overexpressed Vector

We used a vector, pCMV6-*Ngfr*-cDNA, containing a full-length mice *Ngfr* cDNA clone purchased from OriGene Technologies, Rockville, MD, USA. After plasmid transformation, extraction and sequencing, we constructed the *Ngfr* cDNA fragment into a pCAGIG plasmid. The restriction enzyme cutting sites we used were *Eco*RI and *Not*I.

#### 2.4.3. Interference Efficiency Verification

We tested the interference efficiency of shRNA in vitro through co-transfecting the *Ngfr*-overexpressed pCAGIG plasmid with *Ngfr*-interference plasmid/control plasmid into HEK293T cells via Lipofectamine^®^ 2000 (Invitrogen, Carlsbad, CA, USA) liposome. Forty-eight hours later, a Western blot was performed to measure the expression of *NGFR* protein (Western blotting system: Mini-protean^®^ Tetra electrophoresis apparatus and matching transfer tank, Bio-Rad (Laboratories, Hercules, CA, USA); Simon automatic Western blot analysis system, ProteinSimple (San Jose, CA, USA). The cells were cultured in 6-well plates, and the transfection and following protein Western blot was repeated in 1 well for each group. The antibody we used was the *NGFR* antibody (Abcam, Cambridge, MA, USA), and the internal reference was β-actin.

#### 2.4.4. Stereotaxic Surgeries and Microinjection

According to the interference efficiency verification experiment, *Ngfr*-sh2 decreased *NGFR* protein expression most effectively. Thereby, we used the virus vector, Hu6-MCS-CMV-EGFP (GV115), which encodes GFP as well, expressing an effective sequence of GGTCGAGAAGCTGCTCAAT, which was generated by the Genechem Co., Ltd. (Shanghai, China). Identified probe locations for animals used in assays were positioned to the dentate gyrus (DG) of the mouse brain hippocampus, according to its relative location with bregma and lambda on the mouse brain atlas by the stereo locator. The injections were administered in a bio-safety cabinet in a specific room.

Experimental equipment: 1 mL syringe, 10 mg/mL sodium pentobarbiturate, electronic balance, alcohol cotton, cotton swab, surgical scissors, forceps, skull drill (STRONG90), microinjector (Precision Instruments, Sarasota, FL, USA), 3% hydrogen peroxide, 75% alcohol, sterilizer PBS, wound stapler, animal antibiotics, electric blanket, etc.

We performed the intraperitoneal injection in the biosafety cabinet in a dedicated room (10 μL/g). We cut the hair on the top of the head of the mice under deep anesthesia and hung their upper jaw teeth on the positioning rod, which was fixed on the positioning instrument. Then the two sides of the positioning rod were pressed against the skull depression above the mouse’s ear, and the three positioning rods were adjusted to fix the mouse’s head. We cut a small hole lengthwise in the center of the mouse’s head to expose the front funnel and the herringbone point and adjusted the height of the positioning rod to make the anterior fontanelle and the herringbone point consistent. In order to locate the dentate gyrus, we fixed two points with dye at 2 mm backward of anterior fontanelle, 1.6 mm to the left and the right, respectively, and drilled holes with skull surgery. We absorbed 2 μL PBS, 1 μL air and 4 μL virus successively and injected 2 μL virus into each hole at a rate of 500 nL/min. After 3 minutes of setting, we slowly withdraw the needle.

#### 2.4.5. Behavioral Tests

Behavioral tests were performed after 2 weeks of recovery. After verifying by perfusion, we took the following behavioral tests on knockdown mice and controls in order, including an elevated plus maze, open field, novel object recognition, object–place recognition, social test, forced swimming, fear conditioning test and prepulse inhibition (PPI). The images were recorded with a video camera (JVC, TK-C9201EC), and behavior analysis was performed through EthoVision XT software (Version 8.0, Noldus Information Technology, Wageningen, The Netherlands). Data were collected from at least three independent experiments. *T*-tests and One-Way ANOVA for independent samples were performed.

In social tests, four juvenile mice (4–5 weeks) were prepared as social mice. The experimental device was a 3-chambered box system separated by 2 clapboards made of transparent acrylic materials, each with a small hole allowing the mice to enter and exit freely. A metal mesh cover was placed on each side chamber. The mice were placed in the middle chamber and allowed to move freely and adapt for 10 min. The mice were then removed and placed in a temporary empty chamber. A social mouse was then placed in a metal mesh chamber on one side, and the experimental mice were placed in the middle chamber. The activities of the mice were recorded within 5 min. Afterward, the experimental mice and social mice were taken out for the next round of experiments. We used the EthoVision XT software to calculate the time of the mice staying in the social mice chamber and the empty metal mesh chamber and the time of the mice exploring and smelling the social mice and the empty metal mesh chamber.

## 3. Results

### 3.1. Association-Study Analysis of the NGFR SNPs with Schizophrenia

We investigated four *NGFR* SNPs (rs1035050, rs575791, rs2072446, rs11466162) in 1010 patients with schizophrenia and 1034 healthy controls. The distributions of the allele and genotype frequencies of the SNPs in *NGFR* are shown in Table 3. The genotype distributions of all four *NGFR* SNPs conformed to the Hardy−Weinberg equilibrium (HWE, *p*-value > 0.05) in the control group, and only two of them (rs2072446 and rs11466162) appeared to deviate from HWE in the case group (χ^2^ < 8).

The results of association analyses showed that the difference in genotype frequency of rs2072446 and rs11466162 was significantly different between the two groups (*p*-value = 0.016, *p*-value = 0.0032). C allele of rs2072446 was associated with an increased schizophrenia risk (OR = 1.23, *p*-value = 0.041) and A allele of rs11466162 was associated with a decreased schizophrenia risk (OR = 0.807, *p*-value = 0.048). No significant linkage disequilibrium between the SNPs and no haplotype was found.

### 3.2. Correlation between NGFR Gene Polymorphisms and MDD

The distributions of the allele and genotype frequencies of four SNPs in *NGFR* among controls and MDD patients are shown in Table 4. We performed the association-study analysis. The genotype distributions of all four *NGFR* SNPs conformed to HWE in both the case and control groups. There was no statistically significant difference, and no haplotype was found to be significantly associated with MDD susceptibility in further analysis.

### 3.3. Ngfr-Knockdown Mice Showing a Trend of Social Avoidance

**Figure 1:**
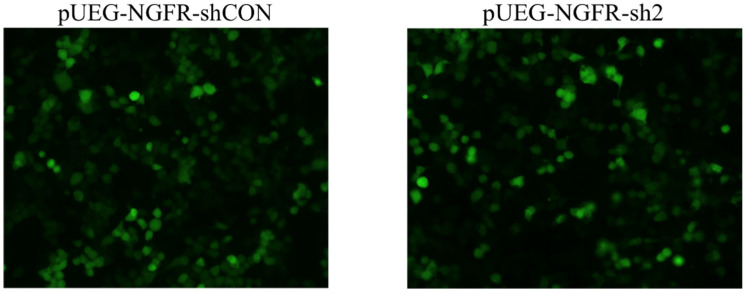
GFP expressed by the transfected cells. The 293T cells were co-transfected with shRNA plasmid and pCAGIG-*Ngfr*-cDNA.

**Figure 2:**
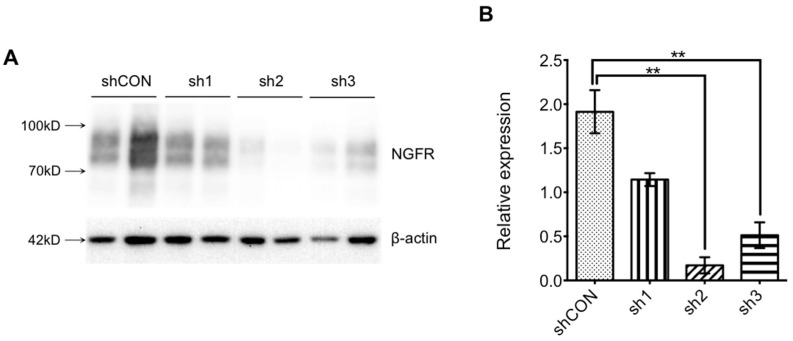
Down-regulation of *NGFR* in 293T cells. (**A**) Immunoblotting of protein extracted from 293T cells co-transfected with shRNA plasmid and pCAGIG-*Ngfr*-cDNA. (**B**) One-way ANOVA of the protein levels after normalization to β-actin; data showed as means ± SEM, ** *p* < 0.01.

**Figure 3:**
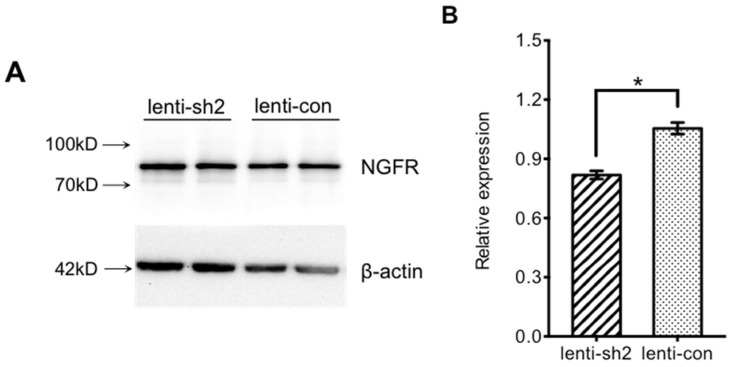
Down-regulation of *NGFR* in mouse hippocampus. (**A**) Lenti-*NGFR*-sh2 or control virus was injected into the dentate gyrus of adult mice. Two weeks after injection, extracts from hippocampus were immunoblotted. (**B**) One-way ANOVA of the protein levels after normalization to β-actin, data showed as means ± SEM, * *p* < 0.05.

**Figure 4:**
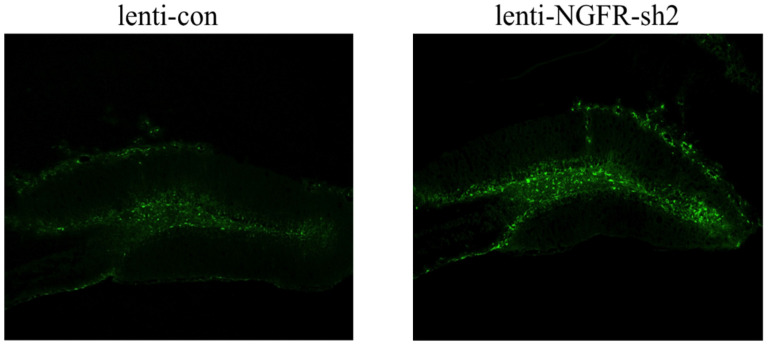
Fluorescence imaging showing the GFP expressed by infected cells in the dentate gyrus of adult mice.

We designed three shRNAs (sh1, sh2, sh3) and a control plasmid co-transfecting 293T cells with a pCAGIG-*Ngfr*-cDNA plasmid for 48 h, respectively. The expression level of *NGFR* protein is shown in Figure 1. As shown in Figure 2, in the interference efficiency verification system, both sh2 and sh3 can knock down the *NGFR* expression level, but sh2 shows a higher efficiency. Therefore, we chose sh2 for lentivirus packaging entrusting Shanghai Jikeiyin Chemical Technology Co., Ltd (Shanghai, China). We injected the virus into the hippocampal dentate gyrus region of the mouse brain. After 2 weeks, we took samples, extracted proteins from the hippocampal tissues of the mice, and performed Western blot detection (*NGFR* antibody: Cell Signaling Technology #8238S). As shown in Figure 3, injection of Lenti-*Ngfr*-sh2 virus successfully down-regulated *NGFR* in mouse hippocampus. We fixed mouse brain tissue by perfusion, performed frozen sections, and observed fluorescence under a microscope to confirm that the virus had been properly injected into the dentate gyrus region of the hippocampus (Figure 4).

**Figure 5:**
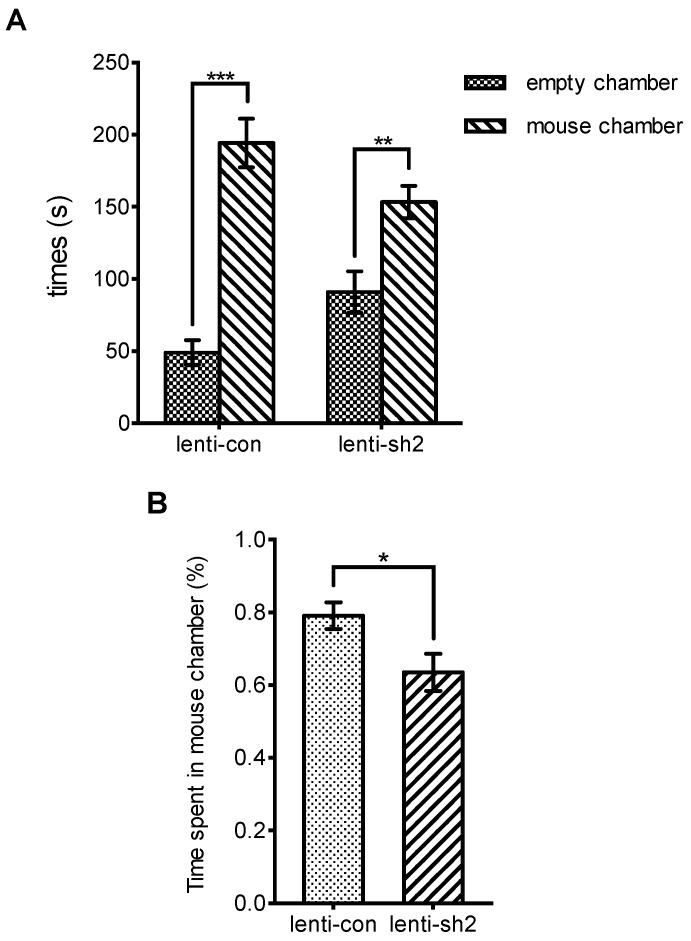
Results of the sociability test. (**A**) Time spent in the empty chamber and the mouse chamber for each mouse. (**B**) Percent of time spent in the mouse chamber was significantly lower for the shRNA mice than control. Results are presented as means ± SEM. *** *p* < 0.001, ** *p* < 0.01, * *p* < 0.05, one-way ANOVA.

In this study, 34 10-week-old C57BL/6 mice were injected with the virus in the hippocampal dentate gyrus. Seventeen mice in the experimental group were injected with Lenti-*Ngfr*- sh2 virus, and seventeen mice in the fossa control group were injected with the Lenti-con virus. All experiments and statistical analyses were conducted in a double-blind manner. In the eight behavioral tests we conducted, including the elevated cross maze experiment, open field experiment, new object recognition experiment, new position recognition experiment, social experiment, forced swimming experiment, conditioned fear experiment, and prepulse suppression experiment, we found significant differences in social behaviors between the experimental and control groups. As shown in Figure 5, we counted the time that mice stayed in the social mouse chamber and the empty chamber, respectively, during the 5-min test period; both groups of mice showed a longer time in the mouse chamber. Comparing the percentages of time spent in the mouse chamber between the two groups, we found that mice in the *Ngfr* shRNA knockdown group spent less time interacting with social mice, showing a trend of social avoidance (Figure 5). No significant differences in other behavioral phenotypes were found.

## 4. Discussion

For the first time, our results indicate that the rs2072446 and rs11466162 polymorphisms in the *NGFR* gene are associated with schizophrenia in the Chinese Han population. We analyzed four *NGFR* SNPS (rS1035050, rs575791, rs2072446, rs11466162) in 1010 schizophrenic patients and 1034 control subjects and found that the genotype frequency of rs2072446 and rs11466162 were significantly different between the two groups (*p*-values were 0.016 and 0.0032, respectively). Our results suggest that the *NGFR* gene is associated with schizophrenia in Chinese Han populations.

The *NGFR* gene encodes the P75 neurotrophic factor receptor, which plays an important role in the regulation of neuronal growth, survival and plasticity. Previous research based on the Armenian Caucasian population [19] and Chinese population [20] revealed the level of *NGFR* in the plasma of schizophrenics was significantly lower than that of healthy controls, suggesting that *NGFR* is linked to the pathogenesis of schizophrenia, especially for the defect of synaptic plasticity. It was reported that the T allele of rs11466155 and T allele of rS2072446 might be risk factors, and the G allele of rs734194 may be a protective factor for schizophrenia in the Armenian Caucasian population [19]. Similar to these findings, in this study, we demonstrated that the genotype frequency distribution of rs2072446 and rs11466162 was significantly different between groups. T allele of rs2072446 may cause the conversion of serine, the 205th amino acid, to leucine in the *NGFR* polypeptide chain. This site is located in a genetically conserved region rich in intracellular serine/threonine, which may be involved in the development of schizophrenia by altering the function of proteins and thus has similar genetic analysis results in different populations. Rs11466162 is located at the 3′UTR of *NGFR,* which may affect psychiatric disorders by cooperating with other SNPs to form undetected haplotypes or regulate protein expressions.

Two recent articles reported higher levels of proBDNF and *NGFR* in depressed patients [17,18]. In the Japanese population, the T allele of rs2072446 was found to be a protective factor for major depression and suicidal behavior [21]. However, in the following association study in the Chinese population, five SNPs, including rs2072446, were found not to be significantly associated with MDD [22]. Rs2072446 and haplotypes rs2072446-rs11466155-rs734194 on the *NGFR* gene was reported to be associated with the efficacy of SSRI antidepressants in Chinese populations [22]. In this research, we also included 610 MDD patients but disproved that the *NGFR* gene was significantly associated with MDD in this Chinese population. These inconsistent results may be due to high heterogeneity among MDD patients and the different ethnic groups we investigated.

Our study suggests that the effects of *NGFR* polymorphisms are not consistent with each other among psychiatric disorders, including schizophrenia, bipolar disorder and depression. As mentioned above, researchers found patients with schizophrenia and bipolar mania have lower levels of serum *NGFR* than healthy people, while MDD patients have higher results, which was led by *NGFR* polymorphisms [17,18,19,20]. It is possible that the *NGFR* gene is directly involved in one or some behavioral phenotypes that take different proportions in different populations with psychiatric disorders, resulting in varying protein levels.

p75NTR knockdown in the hippocampus may affect cell death, survival, and differentiation by interacting with all kinds of TRK receptors, sorting proteins and NOGO receptors. For example, as a receptor for proBDNF, *NGFR* regulates neuronal death by co-acting with coreceptor sorting proteins to activate apoptotic signals. It was reported that chronic stress results in activation of neurodegenerative signaling of proBDNF/*NGFR*/sorting proteins (elevated levels of proBDNF, *NGFR*, and sorting proteins) and reduction in TrkB (inhibited BDNF/TrkB cell survival signals) in the neocortex and hippocampus of rodents. The imbalance between these two opposing pathways may be involved in the pathogenesis of depression and neurodegeneration during stress [23]. Clinical studies on serum *NGFR* protein levels in patients also suggest the potential role of proBDNF/*NGFR* signaling in the pathogenesis of depression [18].

Present behavioral research on *Ngfr* is mainly based on two gene knockout m models, *Ngfr*^exon III−/−^ (carry ing exon III-targeted mutations but still expressing the *NGFR* short protein isoform receptor via alternative splicing [27]) and *Ngfr*^exon IV−/−^ (complete inactivation of *NGFR* [28]). Neither of them has shown consistent behavioral results in *Ngfr*-knockout mice [25,29]. This may be due to the differences in the category background, gender, age and experimental environment of the experimental mice [30]. For example, considering gender factors, Puschban et al. studied the behavioral phenotypes of male and female *Ngfr*^exon IV−/−^ mice and found Sex-specific behavior differences in three behavior tests [28]. The adult lentivirus injection knockdown method used in this paper did not affect the normal *NGFR* protein expression from mouse embryo to adult development. In contrast with previous studies, after eight behavioral tests, we only found that *Ngfr*-knockdown mice showed signs of social avoidance. Due to the different genetic models, whether there is a contradiction still needs to be discussed in the future. The phenotype of social disorder is associated with autism, negative symptoms of schizophrenia, depression, etc. Previous behavioral studies of the *Ngfr* gene in mice did not find similar situations. The results of this work also provide new evidence for the function of this candidate gene.

There are two main limitations in our study. One is the relatively small sample size, which may be the reason for the failure to completely repeat the result of previous research. The other is that we only tested the behavioral phenotypes of knockdown adult male mice and further refined the study by considering differences brought by genders and upregulated *NGFR* protein expression is in need. In the future, more SNPS involved in psychiatric disorders need to be included in the research. Inconsistent results of serum *NGFR* levels suggest that the overexpress test is also in need of further studies. Overexpress polymorphisms of the *NGFR* gene can be a potential way.

## 5. Conclusions

In this study, we demonstrated a significant association between the *NGFR* gene (rs2072446 and rs11466162) and schizophrenia. We investigated the role of *NGFR* in the behavioral phenotype of adult mice by injecting lentivirus in the hippocampal DG region to knock down the protein level and found the abnormal behavior of social avoidance in adult mice, which provided evidence for the *NGFR* gene to be involved in the pathogenesis of psychiatric disorders probably through proBDNF/*NGFR* signals.

## Figures and Tables

**Table 1:** *NGFR* SNPs analyzed in this study.

	SNP ID ^a^	Chromosome ^b^	Location	Polymorphisms ^c^
*NGFR*	rs1035050	17:49486650	promoter	T/C
	rs575791	17:49497393	intron	A/G
	rs2072446	17:49510457	missense	C/T
	rs11466162	17:49513533	3 Prime UTR Variant	G/A

Note. ^a^ According to the dbSNP database, ^b^ the SNP Chromosome positions are based on the NCBI human genome build GRCh38; ^c^ The allele under the slash is the minor allele.

**Table 2:** *NGFR* shRNA and control sequences.

*Ngfr*-sh1-F.	GATCCCGGGCCTTGTGGCCTATATTCTCAAGAGAAATATAGGCCACAAGGCCCTTTTTT
*Ngfr*-sh1-R	CTAGAAAAAAGGGCCTTGTGGCCTATATTTCTCTTGAGAATATAGGCCACAAGGCCCGG
*Ngfr*-sh2-F	GATCCCGGTCGAGAAGCTGCTCAATTTCAAGAGAATTGAGCAGCTTCTCGACCTTTTTT
*Ngfr*-sh2-R	CTAGAAAAAAGGTCGAGAAGCTGCTCAATTCTCTTGAAATTGAGCAGCTTCTCGACCGG
*Ngfr*-sh3-F	GATCCCGCATCCAGAGAGCTGACATTTCAAGAGAATGTCAGCTCTCTGGATGCTTTTTT
*Ngfr*-sh3-R	CTAGAAAAAAGCATCCAGAGAGCTGACATTCTCTTGAAATGTCAGCTCTCTGGATGCGG
shCON-F	GATCCCGTTCTCCGAACGTGTCACGTTTCAAGAGATGCACTGTGCAAGCCTCTTTTTT
shCON-R	CTAGAAAAAAGAGGCTTGCACAGTGCATCTCTTGAAACGTGACACGTTCGGAGAACGG

**Table 3:** Allele and genotype distribution of *NGFR* gene polymorphisms between SCZ patients and healthy participants.

SNP		Genotype Frequency			χ^2^	*p* Value ^a^	Allele Frequency		χ^2^	*p* Value ^a^	Odds Ratio (95%CI)	HWE *p* Value
		CC	CT	TT			C	T				
rs1035050	SCZ	570 (0.576)	362 (0.366)	57 (0.058)	1.01	0.605	1502 (0.759)	476 (0.241)	0.99	0.320	0.929 (0.803–1.07)	0.96
	Control	613 (0.596)	364 (0.354)	52 (0.051)			1590 (0.773)	468 (0.227)				0.83
		AA	AG	GG			A	G				
rs575791	SCZ	609 (0.604)	352 (0.349)	48 (0.048)	1.46	0.482	1570 (0.778)	448 (0.222)	1.40	0.237	0.914 (0.787–1.06)	0.75
	Control	645 (0.626)	344 (0.334)	41 (0.040)			1634 (0.793)	426 (0.207)				0.56
		CC	CT	TT			C	T				
rs2072446	SCZ	808 (0.813)	169 (0.170)	17 (0.017)	8.23	**0.016**	1785 (0.898)	203 (0.102)	4.17	0.041	1.23 (1.01–1.49)	0.022
	Control	784 (0.768)	224 (0.219)	13 (0.013)			1792 (0.878)	250 (0.122)				0.50
		AA	AG	GG			A	G				
rs11466162	SCZ	13 (0.013)	143 (0.142)	853 (0.845)	11.50	**0.0032**	169 (0.084)	1849 (0.916)	3.92	0.048	0.807 (0.652–0.998)	0.015
	Control	6 (0.006)	197 (0.192)	824 (0.802)			209 (0.102)	1845 (0.898)				0.11

^a^ Pearson’s *p*-value, significant *p* (<0.05) values are in bold. HWE: Hardy–Weinberg Equilibrium. SCZ: schizophrenia.

**Table 4:** Allele and genotype distribution of *NGFR* gene polymorphisms between MDD patients and healthy participants.

Scheme 2		Genotype Frequency			χ^2^	*p* Value ^a^	Allele Frequency		χ^2^	*p* Value ^a^	Odds Ratio (95%CI)	H-W *p* Value
		CC	CT	TT			C	T				
rs1035050	MDD	361 (0.605)	201 (0.337)	35 (0.059)	0.83	0.661	923 (0.773)	271 (0.227)	0.00082	0.977	1.00 (0.846–1.19)	0.32
	Control	613 (0.596)	364 (0.354)	52 (0.051)			1590 (0.773)	468 (0.227)				0.83
		AA	AG	GG			A	G				
rs575791	MDD	349 (0.589)	216 (0.364)	28 (0.047)	2.36	0.308	914 (0.771)	272 (0.229)	2.27	0.132	0.876 (0.737–1.04)	0.46
	Control	645 (0.626)	344 (0.334)	41 (0.040)			1634 (0.793)	426 (0.207)				0.56
		CC	CT	TT			C	T				
rs2072446	MDD	479 (0.800)	114 (0.190)	6 (0.010)	2.26	0.324	1072 (0.895)	126 (0.105)	2.19	0.139	1.19 (0.946–1.49)	0.79
	Control	784 (0.768)	224 (0.219)	13 (0.013)			1792 (0.878)	250 (0.122)				0.50
		AA	AG	GG			A	G				
rs11466162	MDD	7 (0.012)	107 (0.182)	475 (0.806)	1.91	0.3844	121 (0.103)	1057 (0.897)	0.0076	0.931	1.01 (0.798–1.28)	0.73
	Control	6 (0.006)	197 (0.192)	824 (0.802)			209 (0.102)	1845 (0.898)				0.11

^a^ Pearson’s *p*-value. HWE: Hardy–Weinberg Equilibrium. MDD: major depressive disorder.
